# Role of Phospholipase D in G-Protein Coupled Receptor Function

**DOI:** 10.3390/membranes4030302

**Published:** 2014-07-03

**Authors:** Lars-Ove Brandenburg, Thomas Pufe, Thomas Koch

**Affiliations:** 1 Department of Anatomy and Cell Biology, RWTH Aachen University, Wendlingweg 2, D-52074 Aachen, Germany; E-Mail: tpufe@ukaachen.de; 2 Department of Pharmacology and Toxicology, Otto-von-Guericke-University Magdeburg, D-39120 Magdeburg, Germany; E-Mail: Thomkoc@gmx.de

**Keywords:** phospholipase D, G-protein coupled receptor, internalization, endocytosis, desensitization, recycling, resensitization

## Abstract

Prolonged agonist exposure of many G-protein coupled receptors induces a rapid receptor phosphorylation and uncoupling from G-proteins. Resensitization of these desensitized receptors requires endocytosis and subsequent dephosphorylation. Numerous studies show the involvement of phospholipid-specific phosphodiesterase phospholipase D (PLD) in the receptor endocytosis and recycling of many G-protein coupled receptors e.g., opioid, formyl or dopamine receptors. The PLD hydrolyzes the headgroup of a phospholipid, generally phosphatidylcholine (PC), to phosphatidic acid (PA) and choline and is assumed to play an important function in cell regulation and receptor trafficking. Protein kinases and GTP binding proteins of the ADP-ribosylation and Rho families regulate the two mammalian PLD isoforms 1 and 2. Mammalian and yeast PLD are also potently stimulated by phosphatidylinositol 4,5-bisphosphate. The PA product is an intracellular lipid messenger. PLD and PA activities are implicated in a wide range of physiological processes and diseases including inflammation, diabetes, oncogenesis or neurodegeneration. This review discusses the characterization, structure, and regulation of PLD in the context of membrane located G-protein coupled receptor function.

## 1. Introduction

Phospholipase D (PLD) is a widely distributed phospholipid-specific diesterase that hydrolyzes phosphatidylcholine (PC) to phosphatidic acid (PA) and choline. PLD is rapidly activated in response to extracellular stimuli, and the generation of PA is considered to mediate many biological functions attributed to PLD and to play an important role in the regulation of cell function and activity. This includes a wide array of cellular responses as calcium mobilization, secretion, superoxide production, endocytosis, exocytosis, vesicle trafficking, glucose transport, rearrangement of actin cytoskeleton, mitogenesis and apoptosis [1]. PLD superfamily members are widely distributed and found in viruses, bacteria, yeast, plants and animals. To date, more than 4000 PLD enzymes have been entered into the NCBI GenBank. The present review focuses on mammalian PLDs and their roles in the G-protein coupled receptor function. For the first time, the PLD activity in human tissue has been described [2]. Two mammalian PLD genes, PLD1 and PLD2, both with two splice variants have been identified [3,4,5]. In line with a role for PLD enzymes in different cellular tasks, PLD1 and PLD2 show a diverse subcellular distribution. PLD1 is found throughout the cell, but primarily localizes to intracellular compartments, including the Golgi apparatus, endosomes, and the perinuclear region [6,7,8]. PLD2 is almost exclusively present at the plasma membrane in lipid raft fractions [9]. Also, PLD1 is found in lipid rafts. The PLD activity appears to be present in nearly all cell types. PLD1 and PLD2 are both robustly expressed in heart, brain, and spleen. PLD1 exhibits low expression in peripheral blood leukocytes and synovial tissue [10], and PLD2 is poorly expressed in liver, skeletal muscle [11] and articular chondrocytes [12]. Both PLD enzymes have been shown to associate with membrane receptors including G-protein coupled receptors (GPCR), receptor tyrosine kinases or integrins, which all mediate signalling of PLD activation. GPCRs constitute a large group of membrane binding receptors known to modulate a wide range of biological responses, including cell growth, differentiation, migration, and inflammatory processes. Extracellular stimuli trigger dissociation of Gα and Gβγ heterotrimeric G proteins. Uncoupled heterotrimer subunits elicit signalling cascades through downstream effector proteins. Many of these pathways elicit functional responses through signalling to PLD in multiple ways. On the other side, the PLD influences GPCR function in many respects. PLD-generated PA affects the GPCR function via modulation of vesicle trafficking, endocytosis and membrane receptor recycling.

## 2. PLD Structure and Regulation of PLD Activity

**Figure 1:**
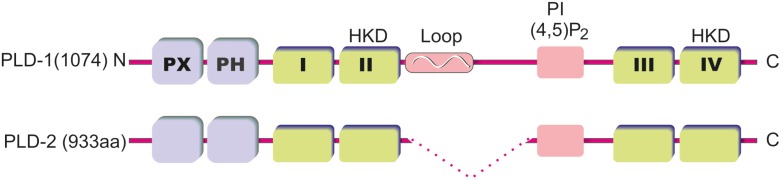
Domain structure of mammalian phospholipase D (PLD) isoforms. The PLD isoforms PLD1 (1074 amino acids) and PLD2 (933 amino acids) contain *N*-terminal PX and PH domains and the highly conserved domains I–IV. The domains II and IV contain HKD sequence motifs that are necessary for catalytic activity. *N*-terminal to domain III is a well conserved basic sequence that binds PI(4,5)P2. PLD1 is distinguished by a loop region that seems to contribute to the regulation of PLD1 activity.

The PLD enzyme is characterized by four conserved regions (I–IV), which form the catalytic core and is flanked by regulatory sequences. Domain II and IV are particularly highly conserved and contain the invariant charged motif designated HKD. The catalytic motif denotes the HxxxxKxD sequence, where the amino acids are histidine (H), any amino acid (x), lysine (K) and aspartic acid (D). The HKD motif responsible for catalytic activity is conserved among all superfamily members. Higher order PLD enzymes are composed of non-conserved regulatory domains. Duplication of the HKD motifs and the repetition of four short motifs, between domains I and II and between domains III and IV, have led to the proposal that eukaryotic PLD genes may be the result of a gene duplication event and that PLD may be a bilobed enzyme [13]. The importance of HKD motif was verified by mutational studies. Substitution of residues in either HKD motif in the PLDs inactivated the enzyme [14]. Other highly conserved regions of the PLD genes are the phox consensus sequence (PX), the plekstrin homology (PH) domain and the PI4,5P_2_ binding site (for the overview about PLD structure, please see Figure 1). The PH domain bind anionic phospholipids such as PI3,4P2 or PI4,5P2 with low specificity and is important for the localization of the protein for palmitoylation at two conserved cysteine residues, as either careful deletion or point mutation causes mislocalization of the proteins [15]. However, deletion of the PH domain demonstrated that it is not required for enzymatic activity and furthermore did not alter the dependence on PI4,5P_2_ for catalysis [16,17]. This finding led to the discovery of the PI4,5P_2_ binding motif. The motif is located between the both HKD motifs and is requisite for catalytic activity [18]. The PX domain binds polyphosphoinositides such as phosphatidylinositol trisphosphate (PI3,4,5P3) with high specificity, and anionic lipids with lower specificity, but this domain has also been implicated in protein interactions with regulatory proteins, including dynamin and Grb2 (Growth factor receptor-bound protein 2) [19,20].

The two PLD isoforms, PLD1 and 2, share 50% sequence homology, mostly at the catalytic domain by a viable length of a conserved loop region *N*-terminal between the HKD and the PI4,5P_2_ motif. PLD1 harbors an extended thermolabile loop prone to proteolytic cleavage [21]. The length of this loop region is variable dependent on the splice variant (PLD1a = 116 aa *versus* PLD1b = 78 aa), while PLD2 does not possess a significant loop region [4]. The loop region has been proposed to function as a possible negative regulatory element, as deletion of this region from PLD1 increased its basal activity threefold [17]. Shortened splice variants of both PLD1 and PLD2 have been identified that compose catalytically inactive enzyme. Expression of these inactive enzymes is observed in different tissues, including the brain, but their function is still unknown [5]. Besides palmitoylation, PLD1, but not PLD2, is multimonoubiquinated at the PH domain in a catalytic and palmitoylation-dependent manner. It was demonstrated that the ubiquitination is important for modulation of protein localization and curbing lipase activity [22].

Mammalian PLD enzymes catalyse a hydrolysis reaction to produce PA. The *in vivo* nucleophile is water, which attacks the diester phosphate group of PC. Both HKD motifs of PLD are needed for the enzymatic action of hydrolysis [14]. Based on biophysical data, the current model of PC catalysis is a two-step mechanism. Initially, the amino-terminal HKD motif is proposed to be protonated on the histidine. Subsequently, PC enters the active pocket of PLD containing both HKD motifs. Next, free choline is released by liberation of the amino-terminal proton, and a PLD-PA intermediate is thought to be formed with the histidine of the carboxy-terminal HKD domain. Finally, the amino histidine re-acquires a proton from a water molecule, leaving the hydroxyl group to attack the PLD-PA intermediate and release PA [11,23]. Interestingly, for mammalian PLDs, short chain primary alcohols are the preferred nucleophile over water (in some cases with more than a 1000-fold preference). This allows the transphosphatidylation reaction to occur at very low concentrations of alcohol [24]. The use of ethanol or 1-butanol allows for a cumulative measurement of PLD activity, as the non-endogenous phosphatidylbutanol or phosphatidylethanol thus formed are relatively stable lipids [25].

The PLD activity is regulated by many factors including small GTPases, kinases or phosphoinositides. The first detected proteins activating mammalian PLD *in vitro* were ARF (ADP ribosylation factor) GTPases [26]. However, to this date the PLD binding site for ARF has not been unambiguously determined. It was assumed that the site might be located near the catalytic domain, because ARF activates *N*-terminally truncated PLD1 and PLD2 [16,27]. The ARF inhibitor Brefeldin A and the expression of dominant negative ARF1 or ARF6 blocked stimulation of PLD [28,29]. The Rho family of GTPases, including RhoA, Cdc42, Rac1, and Rac2, directly activates mammalian PLD. RhoA, Cdc42, and Rac1 selectively activate PLD1 [27]. However, a recent report suggests that Rac2 may activate PLD2 via a previously uncharacterized mechanism [30]. ARF and Rho family GTPases synergize to significantly increase PLD1 activity beyond an additive response. Pretreatment of *Clostridium botulinum* C3 toxin or *Clostridium difficile* toxins, all of which inactivate Rho proteins, blocks PLD activation [31]. Many studies have shown the regulation of PLD activity by protein kinase C (PKC). PKC activating factors including calcium ionophores and phorbol esters such as phorbol 12-myristate-13-acetate (PMA), which is a stable analogue of diacylglycerol (DAG), are very potent activators of PLD. PKC inhibitors, such as staurosporine and calphostin C, block PLD activation [32,33]. The regulation of PLD by PKC appears most likely to involve direct interaction, phosphorylation and possibly other indirect mechanisms. In cells, the classic PKC isoforms α, β and γ stimulate PLD1 and PLD2 activity downstream of PLC activation. PKCα phosphorylates PLD1 and PLD2 at serine and threonine residues, but activation is not phosphorylation-dependent [34,35]. For the PLD1, the PKC binding domain was determined at the amino-terminus [36]. A meaningful overview about the complex regulation of PLD activation by PKC is summarized by McDermott and colleagues [37]. As mentioned above, mammalian PLDs have a PI4,5P_2_ binding domain. Therefore, PLD activity can be stimulated by PI4,5P_2_ and both PLD1 and 2 enzymatic activities are dependent upon PI4,5P_2_ [4,38]. Also, PIP3 activates PLD with similar potency to PI4,5P_2_, but with reduced efficacy [4].

To study the PLD function, the most utilized class of molecule over the past 20 years has been primary alcohols (e.g., *n*-butanol). This is supplemented by overexpression of catalytically active or inactive forms of either PLD1 or PLD2 *in vivo*, or employed RNAi for the individual isoforms in an effort to discern discrete roles for PLD1 and PLD2. Recently, also deficient mice for studies have been available [39,40]. Nevertheless, to assess the therapeutic potential of the inhibition of PLD1 or PLD2, the genetic and biological data must be verified with a small molecule inhibitor. For decades, the quality of the inhibitors was not satisfactory. Whereas the first generation of PLD inhibitors such as calphostin C [41] or curcumin [42] did not distinguish between PLD1 and 2 or also inhibit other signal pathways, the second generation with halopemide showed a clearly higher affinity, but also did not differ between isoforms [43]. Since 2009, the development of isoform-specific PLD inhibitor is driven. Today, highly potent PLD selective inhibitors are available that can be used in animal experiments. The review from Selvy *et al.* [11] described the development of VU0359595 as 1700-fold PLD1-selective inhibitor and VU0364739, a 75-fold PLD2-selective inhibitor. In addition to cellular effects, the inhibitors can be used to investigate the systematic PLD effects in whole organisms.

## 3. GPCR Mediated PLD Signaling

**Figure 2:**
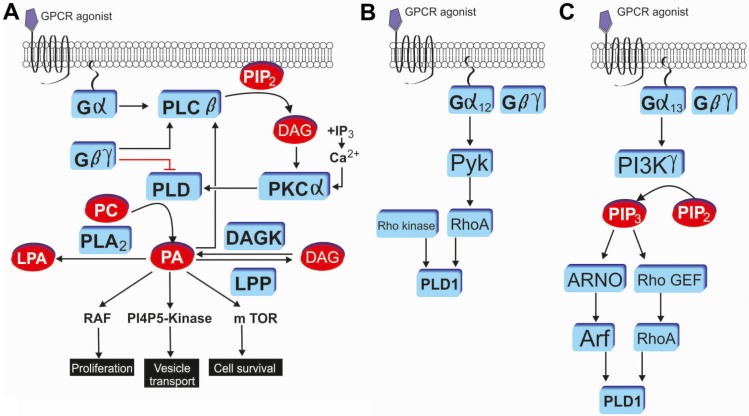
G protein coupled receptor induced PLD activation and phosphatidic acid (PA) mediated signaling through Gαq and protein kinase C (**A**); Gα12 and RhoA (**B**); and Gα13 and Arf (**C**). Modified from Selvy *et al.* [11].

Signal-dependent activation of PLD enzymes has been demonstrated in numerous cell types for many GPCRs. Both PLD isoforms respond to the GPCR activation [44,45]. For example, this includes GPCRs such as μ-opioid [46], cannabinoid type 1 [47], formyl peptide [48] or D2 dopamine receptors [49]. It has been shown that both PTX-insensitive and PTX-sensitive G proteins are involved in stimulation of PLD by GPCRs [50,51]. Remarkably, the GPCR-induced PLD activation is mediated by distinct mechanisms. At first, Gα stimulates PLCβ hydrolysis of PI(4,5)P_2_, producing DAG and IP3. IP3 and DAG synergistically activate PKCα, which in turn bimodally activates PLD [11]. Dissociated Gβγ also activates PLCβ, to indirectly activate PLD in a PKC-dependent manner. On the other hand, Gβγ subunit of the heterotrimer can directly inhibit PLD activity via interactions through the PLD catalytic domain. Gβγ interaction disrupts both basal and ARF stimulated activity [52,53]. Second, the Gα_12/13_ class of heterotrimers activates PLD in a small GTPase-dependent manner. Gα_12_ activates RhoA via Pyk2, a focal adhesion tyrosine kinase, which directly stimulates PLD1 activity. Third, Gα_13_ activates the γ subtype of PI3K to generate PIP3. Upon PIP3 binding, ARNO and Rho GEF trigger GDP for GTP exchange on Arf and RhoA, respectively. These activated small GTPases then directly activate PLD [54] (for the overview about GPCR mediated PLD signaling please see Figure 2).

Interestingly, our own investigations revealed also a direct interaction between μ-opioid receptor and PLD2 [46]. Using a yeast two-hybrid technique, we identified the PX domain in the NH_2_ terminus of the PLD2 to be the important site for the interaction with the COOH terminus of the μ-opioid receptor. The results showed that only PLD2 and not PLD1 was associated with the μ-opioid receptor. A direct association of the PX domain in PLD2 was also shown for other receptors (e.g., epidermal growth factor, platelet-derived growth factor) [55,56]. Interestingly, our results demonstrated an increase of interaction between PLD2 and receptor after agonist treatment and that the PLD2 activation was ARF-dependent. However, for coimmunoprecipitation of μ-opioid receptor and ARF, the presence of PLD2 seemed to be required. Therefore, it is reasonable to assume that ARF binds directly to PLD2 rather than to μ-opioid receptor, but it cannot be excluded that a conformational change of the receptor in the μ-opioid receptor-PLD2 signaling complex is necessary to facilitate ARF binding to the receptor [46].

PLD is a major source of PA generated by cell surface receptor-mediated signaling pathways. PA can be further metabolized to the GPCR agonist LPA (lysophosphatidic acid) by phospholipase A2 and to DAG by phosphatidate phosphohydrolase. However, GPCR-induced DAG production seems to mainly result from PIP_2_ breakdown by PLC [57]. PA directly binds to cellular proteins, such as Raf-1 kinase, protein phosphatase 1, and mTOR, and can affect both cellular localization and activity of these proteins. For the Raf-1 kinase (which is activated by Ras), the recruitment to the plasma membrane requires a direct interaction with PA [58]. Raf-1 (c-Raf) kinase is a proto-oncogene and part of the ERK1/2 pathway as a MAP kinase kinase kinase (MAP3K) that functions downstream of the Ras subfamily of membrane associated GTPases [59]. For the protein phosphatase 1, PA is a potent and selective inhibitor [60]. mTOR (mechanistic target of rapamycin) regulates cell growth, cell proliferation, cell motility, cell survival, protein synthesis, and transcription and belongs to the phosphatidylinositol 3-kinase-related kinase protein family [61]. It is proposed that the responsiveness of mTOR/TOR to PA evolved as a means for sensing lipid precursors for membrane biosynthesis prior to doubling the mass of a cell and dividing [62]. PLD activity also contributes to key events in the oncogenic process including growth signaling, gatekeeper override, suppression of apoptosis, and metastasis [11]. However, a recent work did not confirm that a PLD induced PA formation is important for a mechanically induced increase of mTOR signaling [63]. In addition, PA is involved in a variety of signaling processes, such as activation of phosphoinositide (PI3K, PIP5K) and protein (Akt, ERK1/2) kinases, calcium mobilization, agonist-induced secretion and actin stress fibre formation, and extracellular signal-regulated kinase (ERK)-driven mitogenesis. Next, PA can activate PI4P5-kinase, the enzyme that produce PI(4,5)P_2_ [64]. In addition, it was shown that the PLD2-phosphatidic acid-DAG pathway is involved in the opioid receptor-mediated activation of p38 MAPK that is essential for μ-opioid receptor endocytosis [65]. However, the effects that are mediated by PA or via generation of LPA are multifaceted and not completely understood.

## 4. PLD Influenced GPCR Functions

PLD enzymes influenced GPCR functions about vesicle trafficking, endocytosis and recycling. The first descriptions showed that an overexpression of PLD enhanced internalization, whereas catalytically inactive mutants of PLD or the inhibition of PLD about primary alcohols inhibited endocytosis of receptors [66]. Depletion using PLD2 siRNA, but not PLD1, blocked agonist-induced endocytosis of the angiotensin II receptor [67]. Interestingly, a recent study showed that PLD1 as well as PLD2 inhibition via siRNA influenced formyl peptide receptor internalization [68]. However, both PLD isoforms modulate the receptor endocytosis by different mechanisms. Mammalian PLD enzymes differentially localize to cellular membranes to direct and indirect induce changes in membrane curvature and fusion that facilitate endocytosis/exocytosis and vesicular trafficking. Whereas the PLD2 is located on the plasma membrane, the PLD1 primarily localizes to intracellular membranes. Upon cell stimulation, PLD1 translocates to plasma membrane and is activated [11].

PLD activation and subsequent PA accumulation facilitates vesicle budding and therefore receptor internalization and recycling. PA is a cone shaped lipid and induces changes in membrane curvature. It activates PI4P5K, which generates PI(4,5)P_2_ and induces translocation of coatomer proteins and proteins involved in vesicle budding, including dynamin (a GTPase involved in endocytosis and membrane scission) and AP180 (a clathrin assembly protein). Recently, PLD was also reported to directly interact with dynamin. This interaction occurred in a GTP-dependent manner, and it was suggested that the PX domain of PLD2 might serve as a GAP for dynamin [69]. Altogether, beside PA induced endocytosis, PLD itself influences vesicle scission by a direct interaction with dynamin. In most cases, the agonist-induced receptor endocytosis is clathrin-dependent and requires PLD activity. The activation of GPCR by agonist resulted in a phosphorylation of intracellular receptor domains by GPCR kinases or second messenger-regulated protein kinases, such as Ca^2^^+^/calmodulin-dependent protein kinase II. After phosphorylation of Ser/Thr residues on the C-terminus of the receptor, β-arrestins are frequently recruited to the plasma membrane, where they accelerate uncoupling of the receptor from the G protein and facilitate receptor endocytosis by serving as scaffolding proteins that bind to clathrin. One of the main regulators of the GPCR endocytosis is β-arrestins. Arrestins also interact with phosphoinositides such as PIP2 and PIP3; this interaction appears to play an essential role in mediating arrestin-promoted endocytosis of GPCRs [70]. The interaction of β-arrestins with endocytotic elements including clathrin and the adapter protein 2 (AP-2) are important for induction of clathrin-dependent GPCR endocytosis [71]. The recruitment of the AP-2 adapter complex is also facilitated by PA-mediated increase of PI(4,5)P_2_ level of the membrane [72,73,74]. Clathrin, dynamin and proteins of the AP-2 adapter complex contain domains that mediate their binding to PI(4,5)P_2_-containing membranes [75]. In addition, several groups reported that PLD2 was required for constitutive internalization of different GPCRs. The siRNA depletion of PLD2 function or the use of alcohol inhibited metabotropic glutamate receptors 1 and 5 endocytosis. Also, the constitutive internalization of the μ-opioid receptor was shown to be dependent on PLD2 [76].

**Figure 3:**
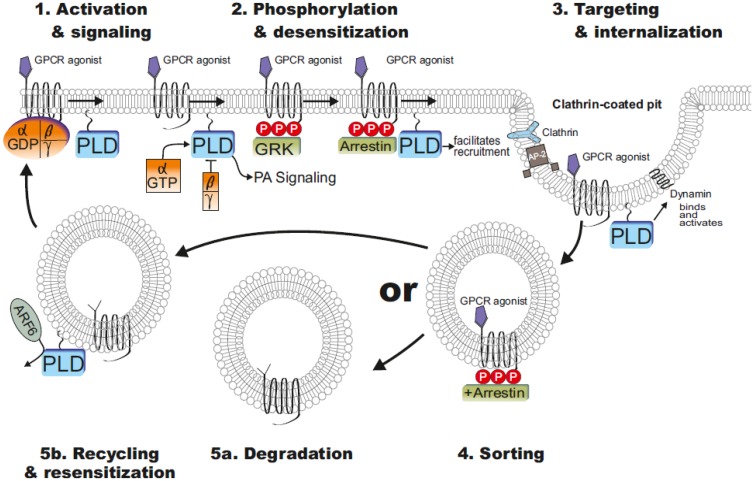
Regulation of G protein coupled receptor (GPCR) trafficking. Agonist binding to GPCRs leads to receptor activation, G protein coupling, and signal transduction including PLD activation. For example, whereas Gα_12/13_ class of heterotrimers or small G proteins such as ARF6 activates PLD, Gβγ subunit of the heterotrimer can directly inhibit PLD (*step* 1). G protein receptor kinases (GRKs) then phosphorylate the agonist-activated GPCR on intracellular domains, initiating arrestin recruitment. Arrestin binding to the receptor inhibits G protein coupling and terminates signaling, a process termed desensitization. PA-mediated increase of PI(4,5)P_2_ level facilitated recruitment of Clathrin, dynamin and proteins of the AP-2 adapter complex (*step* 2). Receptor/arrestin complexes are then targeted to clathrin-coated pits, where arrestin forms a multicomponent complex with clathrin, adapter protein-2 (AP-2), and phosphoinositides, resulting in receptor internalization. PLD directly interact with dynamin and it was suggested that PLD2 might serve as a GTPase activating protein (GAP) for dynamin (*step* 3). Internalized GPCRs are sorted (*step* 4) to degradation (*step* 5*a*) or recycling/resensitization (*step* 5*b*) compartments. ARF6 appears to be involved in the PLD mediated recycling. For details please see the paragraphs. Modified from Moore *et al.* [70].

Internalized receptors can be sorted to a degradative pathway and recycling pathway returning reactivated receptors to the plasma membrane [77]. Internalization, desensitization and receptor trafficking are the predominant mechanisms that control GPCR signaling. Thus, endocytosis serves multiple functions in regulation of GPCR signaling including signal termination, propagation, and receptor resensitization. Trafficking of internalized GPCRs from endosomes to lysosomes and consequent receptor degradation is also an important process that terminates receptor signalling [78]. Figure 3 shows an overview about regulation of GPCR trafficking. The postendocytic fate of receptors is clearly influenced by C-tail domains, binding partners and/or phosphorylation status [70]. In addition to endocytosis, there is evidence that PLD or PA influenced receptor recycling. In particular, ARF6 appears to be involved in the PLD mediated recycling. It was reported that ARF6 is important for recycling of membrane proteins back to the plasma membrane [79]. The inhibition of ARF6 function about dominant negative ARF6 mutants resulted in a decrease of PLD2-mediated μ-opioid receptor recycling [80]. ARF6 and PLD2 seems to be associated with the endosomal membrane [81], but also the PLD1 is activated by ARF6 to facilitate exocytosis [82]. The generation of PA could lead to changes in membrane curvature that might facilitate vesicle fission or fusion [83]. Beside this, small Arf6 GTPase activating proteins ACAP1 and ACAP2 are regulated by PA [84]. In summary, numerous studies have demonstrated that agonist-induced GPCR endocytosis contributes to resensitization of signal transduction by receptor dephosphorylation and recycling to the plasma membrane [85,86,87,88]. The hypothesis is that the GPCR dephosphorylation and recovery from the desensitization requires endocytosis [70]. For the μ-opioid receptor, it has been demonstrated that the endocytotic efficacies of various opioid drugs are negatively correlated with their ability to cause receptor desensitization [86]. In support of this receptor recycling theory, opioid drugs with high endocytotic efficacies induced less opioid tolerance than non-internalizing agonists in rats [89]. These findings support the hypothesis that μ-opioid receptor endocytosis counteracts the development of opioid receptor desensitization and tolerance [86]. However, more recent studies have found that endocytosis seems to be not required for either recovery from desensitization or dephosphorylation of μ-opioid receptor [90,91,92]. It remains to note that the necessity for endocytosis and recycling to dephosphorylate some GPCRs presumably depends on the affinity of arrestins for the agonist occupied receptor. In fact, the affinity of the β-arrestin 2 to μ-opioid receptor is relatively weak, whereas GPCRs with strong β-arrestin 2 interaction such as angiotensin II receptors still need endocytosis to dissolve β-arr2-receptor interaction and uncover the phosphorylation sites for phosphatases [70].

## 5. Pathophysiological Implications of PLD

The systematic investigation of PLD as a therapeutic target has just started in the past few years. Recent reports suggest the high therapeutic potential of PLD inhibition in Alzheimer’s disease [93,94,95], stroke [96] and cancer treatment [97,98]. For Alzheimer’s disease, an increased PLD activity was found in post-mortem brains of Alzheimer patients [99] and in a mouse model of Alzheimer’s disease. Interestingly, PLD2 ablation via gene targeting rescues memory deficits and confers neuronal protection in a mouse model of Alzheimer’s disease despite a significant amyloid β (Aβ) load [93]. In addition, several publications showed an involvement of PLD in Aβ species signaling [95,100]. Furthermore, our *in vitro* results provide evidence for PLD-dependent Aβ1-42 internalization as well as ERK1/2 phosphorylation and the involvement of PI-3-kinases in Aβ1-42-induced formyl peptide receptor like-1 (FPRL1) activation [95]. The involvement of the GPCR FPRL1 in Aβ1-42-induced cell activation was supported by further studies [101,102,103]. Altogether, these studies clearly show that PLD or PLD-mediated PA production could be involved in the development of the Alzheimer’s disease. Therefore, the discovery of highly potent and selective PLD inhibitor or PLD knockout mice will facilitate target validation in animal models of disease. One could speculate if PLD inhibition could also be relevant for mechanically driven disease like osteoarthritis.

## 6. Conclusions and Future Perspectives

During the last decades, a high research effort was made to understand the functions and role of the PLD in the context of membrane function. It has been shown that the PLD contributes to a variety of mechanisms to modulate GPCR signalling or function. The numerous protein–protein and protein–lipid interactions of the PLD emphasize the key position of the enzyme in the temporal and spatial dynamic organization of phospholipid signalling and GPCR function. However, several questions remain unanswered and require further investigations. Future investigations might focus on the possible changes of the PLD-receptor interaction in the context of various diseases and to search for further interaction with other small G-proteins or signal pathways. Thus, it seems reasonable to suggest that in the next years some expected exciting and encouraging results about PLD as a therapeutic target could be expected. In addition, the possible role of PLD mediated endocytosis counteracting the development of opioid tolerance in the context of pain or addiction could be an interesting intervention points for the therapy of opioid addiction and tolerance. The complex function of the PLD enzyme clearly shows how biological systems control the activity of membrane receptors to regulate a diversity of cellular processes. In conclusion, the future studies dealing with the mentioned topics will lead to a more complete understanding of PLD enzymes and GPCR function and regulation.
